# p‐Cresol and p‐Cresyl Sulphate Boost Oxidative Stress: A Systematic Review of Recent Evidence

**DOI:** 10.1111/bcpt.70065

**Published:** 2025-06-18

**Authors:** Rinvil Renaldi, Tjhin Wiguna, Antonio M. Persico, Andi Jayalangkara Tanra

**Affiliations:** ^1^ Doctoral Program, Faculty of Medicine Hasanuddin University Makassar Indonesia; ^2^ Child and Adolescent Division, Department of Psychiatry, Faculty of Medicine Hasanuddin University Makassar Indonesia; ^3^ Department of Psychiatry, Faculty of Medicine Hasanuddin University Makassar Indonesia; ^4^ Child and Adolescent Division, Department of PsychiatryFaculty of Medicine University of Indonesia Jakarta Indonesia; ^5^ Department of Psychiatry dr. Cipto Mangunkusumo General Hospital Jakarta Indonesia; ^6^ Department of Biomedical, Metabolic and Neural Sciences University of Modena and Reggio Emilia Modena Italy; ^7^ Child & Adolescent Neuropsychiatry Program Modena University Hospital Modena Italy

**Keywords:** autism spectrum disorder, chronic kidney disease, gastrointestinal microbiome, Parkinson disease, uremic toxins

## Abstract

Recent studies have emphasized the significant role of p‐cresol and its conjugated form, p‐cresyl sulphate (PCS), in enhancing oxidative stress, leading to potential detrimental effects on various biological systems. Both p‐cresol and PCS contribute to increased production of reactive oxygen species (ROS), which can result in tissue damage, inflammation and a cascade of physiological abnormalities. Elevated p‐cresol levels have been associated with greater clinical severity in autism spectrum disorder, correlating with more severe behavioural manifestations and a history of regression. This systematic review explores the recent evidence on how these compounds promote oxidative stress and their impact on different health conditions. This review also addresses the involvement of p‐cresol and PCS in conditions such as chronic kidney disease, Parkinson's disease and other neurodegenerative disorders, where oxidative damage contributes to disease progression. Furthermore, this review highlights the need for further research to understand the precise mechanisms by which p‐cresol and PCS modulate oxidative stress and their potential as biomarkers for clinical diagnosis and disease management.

**Summary:**

- This focused review systematically summarizes recent evidence that oxidative stress plays an important role in the damage of biological systems produced by two uremic toxins, p‐cresol and its conjugated form, p‐cresyl sulphate (PCS).
- p‐cresol coming from environmental sources or produced by some gut bacterial strains, modulates various conditions, like chronic kidney disease, Parkinson's disease and autism spectrum disorder, among others.
- Oxidative damage and inflammation seemingly contribute to disease onset, progression and/or severity.
- The exact mechanism by which p‐cresol and PCS promote oxidative stress, their influence on disease trajectory and their potential role as biomarkers merit further investigation.

## 1 Introduction

Oxidative stress (OS) is a condition characterized by the imbalance of the production and degradation of reactive oxygen species (ROS) or reactive nitrogen species (RNS) leading to disruption of redox signalling and cellular or molecular damage. These molecules have high reactivity and are products of oxygen or nitrogen metabolism. Examples of ROS and RNS are free radicals such as superoxide radical (O_2_^−^), hydroxyl radical (OH•), nitric oxide (NO) and non‐free radicals like hydrogen peroxide (H_2_O_2_) and peroxynitrite (ONOO^−^). ROS can oxidize the mitochondrial complexes involved in the electron transport chain, reducing the efficiency of oxidative phosphorylation and thus hampering ATP production. Elsewhere, they can also interfere with many enzymatic activities (methionine synthase is a paradigmatic example) and with lipid membrane fluidity, directly or indirectly affecting several metabolisms as well as receptor function [1]. Additionally, ROS are produced in other cellular compartments, such as the endoplasmic reticulum and peroxisomes, where they play roles in cellular signalling and metabolism but can also contribute to cellular dysfunction when their levels exceed the cell's capacity for detoxification [2, 3].

Mitigation of oxidative damage is achieved through a variety of enzymatic and non‐enzymatic antioxidant mechanisms. The primary antioxidant enzymes, namely superoxide dismutase (SOD), catalase (Cat) and glutathione peroxidase (GPx), possess the capability to neutralize reactive oxygen species (ROS). Beyond these enzymatic antioxidants, ROS can also be mitigated by non‐enzymatic molecules that exhibit free radical scavenging properties, including vitamins, melatonin and glutathione (GSH). In circumstances where antioxidant defences are insufficient to neutralize ROS, these reactive species persist within the biological system for extended periods, leading to the oxidation of vulnerable biomolecules, which in turn disrupts cellular functions and initiates pathological processes. The detrimental disparity between antioxidant capacity and free radical concentrations, culminating in oxidative stress, is hypothesized to play a significant role in the pathophysiological development, progression and severity of various conditions, including hypertension, atherosclerosis, chronic kidney disease, diabetes mellitus, arthritis, neurodegenerative diseases, cancer, and more recently, neurodevelopmental disorder. As evidenced by numerous studies, autism spectrum disorder (ASD) has been associated with elevated oxidative stress through multiple mechanisms, including the toxic accumulation of reactive oxygen species, aberrant lipid peroxidation, alterations in protein structures and diminished antioxidant capacity [2, 4]. Furthermore, the brains of children exhibit heightened susceptibility to oxidative stress compared to adults due to their comparatively lower antioxidant defences and detoxification capabilities [5]. Research indicates that children diagnosed with ASD demonstrate significantly reduced levels of glutathione (GSH)/oxidized glutathione (GSSG) ratio, methionine, cysteine, vitamins B9, B12, E, D, S‐adenosylmethionine/S‐adenosylhomocysteine and calcium concentrations [6]. Efforts aimed at enhancing antioxidant capacity to ameliorate oxidative stress through supplementation with antioxidants such as vitamin C, coenzyme Q10, sulforaphane, N‐acetylcysteine, cocoa and melatonin may yield improvements in prognosis by mitigating irreversible damage within the ASD‐afflicted brain and alleviating the symptoms experienced by children with ASD [4, 5, 7].

p‐Cresol and its conjugated derivative, p‐cresyl sulphate (PCS), uremic toxins, are a member of the cresol group of organic aromatic compounds, has significant toxicological implications for humans. It is naturally produced through the photo‐oxidation of toluene, an environmental chemical, which can lead to human exposure via inhalation or skin contact. Another major source of p‐cresol is gut bacteria, which generate it through the tyrosine fermentation pathway. This pathway, driven by bacteria such as *Clostridium difficile* , *C. scatologenes* and certain *Lactobacillus* species, becomes particularly significant under conditions of microbial overgrowth or dysbiosis, as frequently occurs in children with ASD [8]. The abundance of L‐tyrosine in the gut resulting from such factors such as slow intestinal transit time, increased gut epithelial turnover, chronically elevated tyrosine intake, excessive dietary protein consumption, or pharmacological inhibition of gastric acid secretion) may further promote microbial synthesis of p‐cresol. Both p‐cresol and PCS exert toxic effects on multiple physiological systems, including the liver, cardiovascular, immune and nervous systems. These effects are mediated by mechanisms such as oxidative damage, mitochondrial inhibition, immune suppression and metabolic disruption. Furthermore, these compounds are linked to chronic diseases like chronic kidney disease and may contribute to conditions such as ASD [8].

There is a positive correlation between elevated urinary p‐cresol levels and clinical severity in children with ASD, especially in those with a history of regression. This suggests that p‐cresol may exacerbate ASD symptoms [9]. A replication study found similar results, indicating that urinary levels of both p‐cresol and PCS, were elevated in ASD. These elevated levels were associated with stereotypic and compulsive/repetitive behaviours and were more pronounced in younger children, particularly those under 8 years old, and those with more severe ASD symptoms [10].

Given that p‐cresol and PCS could modulate autism severity and acting through several mechanisms, including the pro‐inflammatory effects of PCS as chemical and toxins could contribute to enhanced oxidative stress in ASD [11], we sought to further examine the impact of p‐cresol and PCS on various biological systems and their contribution to oxidative stress. Therefore, this systematic review aims to consolidate and summarize the current evidence regarding the association of p‐cresol and PCS with oxidative stress focusing on recent developments of the literature published during the last 5 years [12, 13, 14].

## 2 Material and Methods

### 2.1 Search Strategy

In this systematic review, a comprehensive literature search was conducted to identify relevant studies published during the last 5 years. The search strategy employed the following keywords: ‘oxidative stress’ AND ‘p‐cresol’ OR ‘cresol’ OR ‘4‐methylphenol’ OR ‘4‐cresol’ OR ‘p‐cresyl sulphate’, specifically within the titles or abstracts of the studies. The search was executed across five databases: PubMed, EBSCOhost, Scopus, ProQuest and Elsevier, covering studies published from January 2020 to December 2024. A detailed list of the keywords used in each database and article type is provided in Table 1.

**TABLE 1:** Search strings used to search each database.

No.	Database	Keywords
1.	PubMed (47)	((‘oxidative stress’[Title/Abstract] AND ‘p‐cresol’[Title/Abstract]) OR ‘cresol’[Title/Abstract] OR ‘4‐methylphenol’[Title/Abstract] OR ‘4‐cresol’[Title/Abstract] OR ‘p‐cresyl‐sulfate’[Title/Abstract]). Article type: Clinical study, Clinical article, Observational study, Observational study veterinary, Randomized controlled trial, Randomized controlled trial veterinary. Publication date: last 5 years
2.	Scopus (48)	TITLE‐ABS (oxidative AND stress) AND (TITLE‐ABS (p‐cresol) OR TITLE‐ABS (cresol) OR TITLE‐ABS (4‐cresol) OR TITLE‐ABS (p‐cresyl‐sulfate) OR TITLE‐ABS (4‐methylphenol)) AND PUBYEAR > 2019 AND PUBYEAR < 2025 AND (LIMIT‐TO (DOCTYPE, ‘ar’))
3.	EBSCOhost (47)	AB oxidative stress AND AB p‐cresol OR AB cresol OR AB p‐cresyl‐sulfate OR AB 4‐cresol OR AB 4‐methylphenol. Full Text; Publication Date: 20200101–20 241 231
4.	ProQuest (12)	abstract (oxidative stress) AND abstract(p‐cresol) OR abstract (cresol) OR abstract(4‐cresol) OR abstract(p‐cresyl‐sulfate) OR abstract(4‐methylphenol). Limits: Full text, Date: From 1January 2020 to 31 December 2024, Source type: Evidence‐Based Medical Resources, Scholarly Journals, Document type: Case Study, Dissertation/Thesis, Evidence Based Healthcare, Report
5.	Elsevier (25)	oxidative stress AND (p‐cresol OR cresol OR 4‐methylphenol OR 4‐cresol OR p‐cresyl‐sulfate). Limits: Date: 2020–2024

### 2.2 Inclusion and Exclusion Criteria

A set of inclusion and exclusion criteria was applied during the selection process for this systematic review. The inclusion criteria were as follows: (1) human studies, primarily cohort studies and case control studies; (2) animal studies, including experimental in vivo studies on animal models; (3) studies published in English, French, Spanish or Italian. The exclusion criteria were as follows: (1) article types, including reviews, case reports, conference abstracts, book chapters, opinion papers, editorials and letters; (2) studies with inaccessible full text; (3) studies with data that could not be extracted despite attempts to contact the corresponding author; (4) studies published in languages other than English, French, Spanish or Italian.

### 2.3 Data Extraction, Study Outcomes and Risk of Bias Assessment

#### 2.3.1 Data Extraction

One reviewer conducted the initial search of the databases to identify the studies pertaining to the topic and filter the results based on inclusion criteria by screening title, abstract or full text. The second reviewer then checked this initial selection. Any disagreement between the two reviewers has been resolved by discussion. The details of information that were extracted from the included article were as follows: (1) authors, year of publication, country; (2) study design; (3) characteristics of study sample; (4) type of exposure/intervention; (5) results. Data have been collected in an Excel spreadsheet.

#### 2.3.2 Study Outcomes

The main outcomes of interest observed in this review were as follows: (1) levels of biomarkers of oxidative stress, such as reactive oxygen species (ROS), malondialdehyde (MDA) and antioxidant enzyme activity (e.g., superoxide dismutase (SOD), catalase (CAT) and glutathione peroxidase (GPx); (2) empirical data about the association of p‐cresol and p‐cresyl‐sulphate to oxidative damage, including mitochondrial dysfunction, lipid peroxidation, or DNA damage. Moreover, association between p‐cresol‐induced oxidative stress and disease conditions, such as chronic renal disease, cardiovascular diseases, autism spectrum disorder and neurodegenerative disorders.

#### 2.3.3 Risk of Bias Assessment

The risk of bias in case–control studies was assessed using the Newcastle–Ottawa Scale (NOS). This tool evaluates three distinct domains: selection, comparability and outcome/exposure, with maximum scores of 4, 2 and 3 points, respectively. Each domain comprises specific components that address various aspects of the study design. The components have been applied in accordance with the protocol established for cohort and case–control studies. Following the evaluation of each domain, a judgement regarding the risk of bias has been assigned, based on the criteria specified by the Newcastle–Ottawa Scale [15].

The risk of bias for in vitro quasi experimental studies has been assessed using the revised Joanna Briggs Institute (JBI) critical appraisal tool for quasi‐experimental studies. The revised JBI critical appraisal tool for quasi‐experimental studies consists of a set of questions to determine the risk of bias or address other factors that are related to the study validity or quality. This critical appraisal tool assesses seven different domains of bias such as temporal procedure bias, selection and allocation bias, confounding factors bias, administration of intervention or exposure bias, outcome assessment, detection and measurement bias and participant retention bias. Following the evaluation of each domain, a judgement regarding the risk of bias was assigned based on the JBI critical appraisal tool for quasi‐experimental studies. Both assessments have been conducted by two independent reviewers, and any discrepancies have been resolved through consensus among all authors [16].

#### 2.3.4 Data Synthesis

Key features of each study, including sample characteristics, intervention details and outcomes, have been systematically extracted. A comprehensive analysis was conducted to identify common patterns and themes across the studies, with a focus on the differential effects of microbiota‐derived metabolites in both in vitro and in vivo models of chronic kidney disease, cardiovascular diseases, autism spectrum disorder and neurodegenerative disorders.

No formal statistical analysis was performed, as this review primarily aims to systematically identify and summarize recent mechanistic studies conducted using diverse experimental models, rather than to aggregate case–control studies for meta‐analysis. The results will be presented as a narrative synthesis, organized by compound and underlying mechanism, with an emphasis on consistent findings and identification of gaps in the current literature.

#### 2.3.5 Registration of the Systematic Review

This systematic review was registered in the International Prospective Register of Systematic Reviews (PROSPERO) on December 13, 2024, with id. CRD42024621293.

## 3 Results

### 3.1 Study Selection, Study Characteristics and Quality Assessment

**FIGURE 1:**
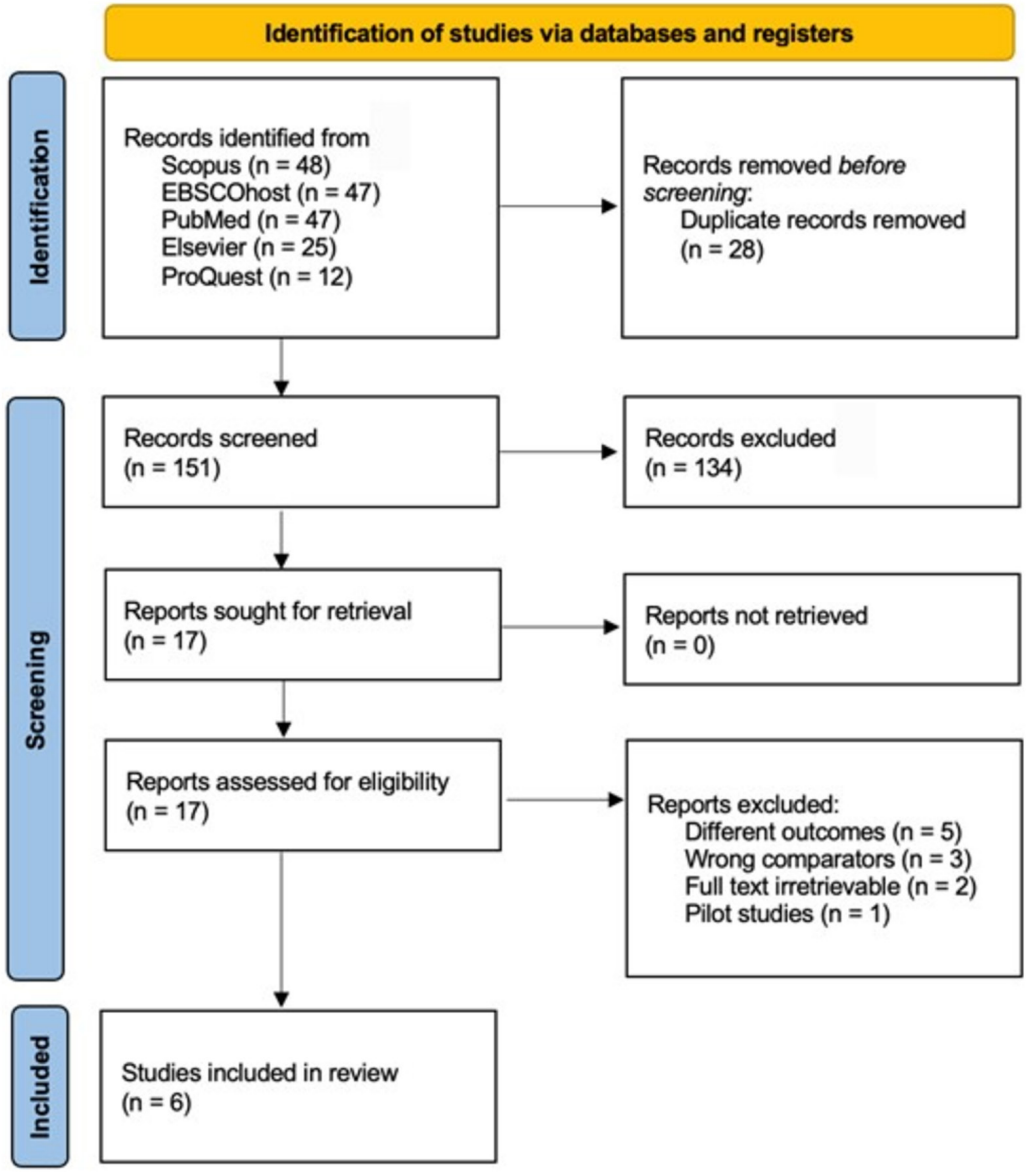
PRISMA flowchart.

We initially identified 179 records through a comprehensive search across five databases: Scopus, EBSCOhost, PubMed, Elsevier and ProQuest. After removing 28 duplicate records, a preliminary screening was conducted based on the titles and abstracts of the remaining articles. Of these, 134 records were excluded according to predefined inclusion and exclusion criteria. This initial screening yielded 17 studies that were deemed eligible for further evaluation at the full‐text level. Subsequently, 11 studies were excluded for the following reasons: five studies investigated outcomes unrelated to oxidative stress, three studies utilized inappropriate comparators (e.g., indoxyl sulphate), two studies had inaccessible full‐text articles, and one study was a pilot study. Ultimately, 6 studies met the inclusion criteria and were retained for this review. A detailed flowchart outlining the systematic search and selection process is presented in Figure 1.

The final selection of six studies consisted of two case–control studies and four in vitro quasi‐experimental studies. These studies were conducted across various countries: two in Brazil, one in France, one in Canada, one in Georgia and one in Taiwan. The studies were published between 2020 and 2022. The eligible study subjects included humans, animals and human‐ or animal‐derived cells. Specifically, one study was conducted on humans, one on mice and four studies involved cell lines, including pheochromocytoma cells (PC‐12), HepaRG cells (metabolically competent human primary hepatoma liver cells), the human endothelial cell line EA.hy926, human monocytic‐like U‐937 cells and mouse 3T3‐L1 adipocytes.

The quality and risk of bias assessments for the six included studies are presented in Supplementary Tables 1A and 1B for case–control and in vitro quasi experimental studies, respectively. Overall, the majority of studies demonstrated good quality.

### 3.2 Study Outcomes

The effects and role of p‐cresol and its derivative PCS in the modulation of oxidative stress remain insufficiently explored. Although numerous studies speculate on the potential mechanisms through which p‐cresol and PCS may induce oxidative stress, most research has primarily identified elevated concentrations of these compounds under oxidative stress‐related conditions, such as neurobehavioral disorders and chronic kidney disease. This systematic review provides recent evidence that p‐cresol and PCS can indeed induce oxidative stress and through this effect may play a significant role in the pathophysiology of various diseases characterized by excessive oxidative stress, including chronic kidney disease (CKD), neurodegenerative and neurodevelopmental disorders [13, 14, 17, 18, 19, 20].

Table 2 provides a detailed summary of the findings from all six studies, which were selected through our rigorous and systematic review process. These studies utilized a diverse range of experimental models, including human subjects, human‐derived cells, mice and mouse‐derived cells, and explored various conditions associated with oxidative stress, such as neurobehavioral abnormalities and chronic kidney disease. The studies employed a broad spectrum of p‐cresol concentrations, ranging from 0.25 to 212 μM. All studies collectively support the conclusion that p‐cresol plays a significant role in the induction of oxidative stress. Specifically, the studies identified several key mechanisms through which p‐cresol and/or its derivative PCS contribute to oxidative stress, including the activation of nicotinamide adenine dinucleotide phosphate (NADPH) oxidase, dysregulation of brain‐derived neurotrophic factor (BDNF) secretion, dopaminergic and serotonergic neurotransmission, increased levels of corticosterone and repressor element 1‐silencing transcription factor (REST) and the induction of extracellular vesicle (EV) formation yielding inflammation through cell–cell communication. Additionally, the studies highlighted potential intracellular cascades underlying the oxidative stress induced by p‐cresol, including the activation of transcription factors such as cAMP response element‐binding protein (CREB) and activating transcription factor 1 (ATF1). Furthermore, one study utilized dichlorofluorescein (DCF) as a marker for assessing oxidative stress in cells, providing a quantitative measure of ROS production.

**TABLE 2:** Summary description and outcome for each of the six studies selected by our systematic search.

No.	Author; year	Study design	Population/subjects	Main results and conclusions
**1**.	**Koppe et al. 2021** [8]	Case–control	Mouse 3T3‐L1 adipocytes treated with p‐cresyl‐sulphate (PCS) 212 μM, to mimic levels encountered in end‐stage chronic kidney disease (CKD) vs KCl used as control. In some experiments, cells underwent pre‐treatment with probenecid, n‐acetylcysteine (NAC), ascorbate, or α‐tocopherol for 2 h prior to PCS treatment	No difference in the amount of living cells and no significant increase of extracellular LDH between cells exposed to PCS for 2 h and 16 h and control. Exposure of PCS caused a transient increase in ROS production that lasted for 4 h. PCS increase production of ROS starting at a concentration of 100 μM (the common amount observed in CKD patients). PCS enters cells through the organic anion transporter 3 (OAT3). The OAT inhibitor probenecid (1 mM) prevents PCS‐induced increase in ROS production. The NADPH oxidase inhibitor apocynin (1 mM) prevent the production of ROS triggered by PCS. Glutathione content in 3T3L1 cells exposed to PCS decreases as much as 47% (*p* < 0.05) compared to control. Pretreatment with antioxidants, including NAC (1 mM), ascorbate (200 μM), or α‐tocopherol (2.5 μM) prevent oxidative stress induced by PCS. **Conclusions:** PCS at the concentration found in end‐stage renal disease patients does not trigger direct cell death, but induces significant oxidative stress through NADPH oxidase activation. Antioxidants prevent oxidative stress induced by PCS in 3T3‐L1 adipocytes.
**2**.	**Sun et al. 2020** [3]	Case–control	Eighty‐eight 10‐week‐old male C57BL/6 mice (68 mice were subjected to unilateral nephrectomy and 20 mice were used as controls. Both groups (20 controls and 20 mice with unilateral nephrectomy) were given various doses of PCS (0, 1, 10 and 100 mg/kg/day i.p. for 7 weeks) for dose‐effect study 32 mice that underwent unilateral nephrectomy were divided into 2 groups that were given PCS vs saline 16 mice were subjected to unilateral nephrectomy and given PCS together with the spherical carbonaceous uremic toxin absorbent AST‐120 (400 mg/kg p.o.)	Chronic exposure to high doses of PCS (100 mg/kg/day) yields only in unilateral nephrectomized mice increased immobility time in the forced swim test and tail suspension test, as well as less time spent in the light compartment in the light/dark box test, and less effective responses in the Morris water maze. PCS reduces microtubule‐associated protein 2 (MAP‐2) content in the prefrontal cortex. PCS impairs brain‐derived neurotrophic factor (BDNF) signalling and serotonin neurotransmission, and increases the production of corticosterone and repressor element‐1 silencing transcription factor (REST) PCS mice show increased levels of malondialdehyde (MDA) and decrease levels of glutathione (GSH) Serum level and prefrontal cortical tissue content of IL‐β1 protein is increased in PCS mice, accompanied by an increase in phosphorylation of p38, c‐Jun N‐terminal Kinase (JNK), and p65. **Conclusions:** PCS produces anxiety‐ and depression‐like behaviours, and impairs spatial memory and learning only in unilateral nephrectomized mice. PCS has negative effects on neuronal cells and neural stem cells, and promotes cell apoptosis causing neurodegeneration, impaired neurogenesis, enhanced oxidative stress, and neuroinflammation in the prefrontal cortex, also through reduced BDNF and serotonin, and increased corticosterone and REST. The PCS‐induced changes can be alleviated by the uremic toxin absorbent AST‐120
**3**.	**Cunha et al. 2022** [9]	In vitro quasi experimental	Peripheral blood samples from 80 patients aged 18–80 at various stages of CKD. External segments of the iliac and renal arteries collected during transplantation procedure from healthy kidney donors and from recipients with CKD. Human endothelial cell line EA.hy926 exposed to normal (0.08 mg/L), uremic (1.75 mg/L), and maximum uremic (2.6 mg/L) PCS concentrations for 24 h,	PCS significantly decreases cell viability (*p* < 0.001) at all concentrations tested, without acting through the cAMP‐responsive element‐binding protein (CREB) or activating transcription factor 1 (ATF1) pathway PCS significantly increases the expression of ATF1 PCS significantly increases the phosphorylation of CREB and ATF1, implying an activation of the CREB/ATF1 pathway PCS significantly increases the transcription of genes controlled by CREB and ATF1, such as ICAM1, PTGS2, and NOX1. **Conclusions:** PCS is capable of activating transcription factors CREB and ATF1 and of inducing CREB/ATF1‐regulated gene expression in endothelial cells. The induction of CREB/ATF1‐regulated genes, like *ICAM1*, *PTGS2*, and *NOX1*, boosts vascular inflammation and oxidative stress, fostering endothelial dysfunction in CKD.
**4**.	**Zhu et al. 2021** [5]	In vitro quasi experimental	HepaRG cells were treated with concentrations ranging between 0 and 2 mM p‐cresol, and for exposure times between 0 and 24 h. After the exposure, all cells are measured in 2′‐’‐dichlorofluorescein (DCF) assay to measure oxidative stress and total cellular glutathione (GSH) assay, and lactate dehydrogenase (LDH) assay to measure cellular necrosis.	24 h exposure to p‐cresol increased DCF formation, decreased total cellular GSH concentration, and increased LDH release starting at 0.25, 0.75 and 0.50 mM p‐cresol, respectively. p‐Cresol was more toxic than other tested uremic toxins (CMPF, indole‐3‐acetic acid, indoxyl sulphate, kynurenic acid, hippuric acid) and also compared to its derivatives PCS and PCG in inducing oxidative stress, glutathione depletion, and cellular necrosis. **Conclusions:** p‐Cresol is capable of inducing oxidative stress, glutathione depletion, and cellular necrosis. P‐cresol is a more potent toxicant compared to other uremic toxins and to its derivatives PCS and PCG.
**5**.	**Favretto et al. 2021** [10]	In vitro quasi experimental	Human endothelial cell line EA.hy926 and human monocytic‐like U‐937 cells were incubated with PCS (2.6 mg/L) for 3 h. Extracellular vesicles (EVs) from cells exposed to PCS were isolated and their effect on endothelial cells not exposed to uremic toxins was assessed.	PCS can induce the formation of extracellular vesicles (EVs) from endothelial cells. A new batch of endothelial cells exposed to EVs formed by PCS‐exposed endothelial cells undergoes: ✓ Decreased adhesion ✓ Increased inflammation due to upregulated VCAM‐1 expression ✓ Increased migration. **Conclusions:** PCS can induce formation of EVs yielding decreased adhesion, increased inflammation, and increased migration of endothelial cells. PCS could promote widespread endothelial damage through EV‐mediated cell–cell communication.
**6**.	**Tevzadze et al. 2021** [11]	In vitro quasi experimental	PC‐12 cells were treated with low‐dose p‐cresol (1 μM) for 5 days. After 5 days, the cells were treated with nerve growth factor (NGF) for 5 days. PC‐12 cells were treated with p‐cresol (1 μM) and/or rizatriptan (1 μM) and/or enkephalin and/or oxytocin (1 μM) for 5 days	Low doses of p‐cresol (1 μM) potentiated NGF‐induced differentiation of PC‐12 cells by increasing BDNF secretion. Low doses of p‐cresol increased the expression of NF subunits in PC‐12 cells, indicating increased neuronal differentiation and structural remodelling. The effects of p‐cresol on BDNF secretion were modulated by opioidergic and serotoninergic compounds, with enkephalin and rizatriptan increasing BDNF secretion, while oxytocin reversed the effects of p‐cresol. **Conclusions:** Low dose p‐cresol can increase BDNF secretion, with enkephalin and rizatriptan promoting and oxytocin antagonizing this effect The PC‐12 cell differentiation promoted by low dose p‐cresol through enhanced BDNF secretion may be interpreted as a possible protective mechanism triggered by oxidative stress.

Abbreviations: AST‐120, an oral spherical carbon adsorbent; BDNF, brain‐derived neurotrophic factor; CKD, chronic kidney disease; CMPF, 3‐carboxy‐4‐methyl‐5‐propyl‐2‐furanpropanoic acid; EA.hy926, a human endothelial cell line used in the experiment; ICAM‐1, intercellular adhesion molecule‐1; GSH, total cellular glutathione concentration; HepaRG, human primary hepatoma liver cell line; KCl, kalium chloride, potassium chloride; LDH, lactate dehydrogenase; MAP‐2, microtubule‐associated protein 2; NAC, N‐acetyl‐cysteine; NADPH, nicotinamide adenine dinucleotide phosphate; NGF, nerve growth factor; NOX‐1, NADPH oxidase 1; OAT, organic anion transporter; p‐CS, p‐cresyl sulphate; PC‐12 cells, rat pheochromocytoma cells; PTGS2, prostaglandin‐endoperoxide synthase 2; ROS, radical oxygen species; U‐937 cell, a human monocytic‐like cell line.

## 4 Discussion

The studies reviewed here provide converging evidence that p‐cresol induces oxidative stress and related pathophysiological effects, making finding from experimental models relevant to the clinical setting. In children with ASD this occurs at p‐cresol concentrations ranging from 0.25 to 212 μM, which span plasma levels recorded in CKD, typically exceeding 100 μM. At these concentrations, plasma levels of p‐cresol in CKD patients are sufficient to contribute to cellular and systemic dysfunction. Several potential mechanisms through which p‐cresol and PCS contribute to oxidative stress have been identified. These mechanisms include the activation of NADPH oxidase, dysregulation of neurotrophic factors (e.g., brain‐derived neurotrophic factor, BDNF) and neurotransmitters (e.g., dopamine and serotonin) and the induction of EV formation able to exert a pro‐inflammatory modulation on distant cells. One of the studies highlighted the use of DCF as a reliable marker for quantifying oxidative stress at the cellular level [21]. These findings highlight the potential of p‐cresol and PCS to contribute to oxidative damage and their involvement in the pathogenesis of various oxidative stress‐related disorders [13, 14, 17, 18, 19, 20].

NADPH oxidase (NOX) enzymes are membrane‐bound multi‐subunit complexes that function to transfer electrons across cellular membranes to molecular oxygen, thereby generating superoxide anions (O_2_^−^) and other ROS, including hydrogen peroxide (H_2_O_2_) and hydroxyl radicals (OH•). These ROS can modify cellular proteins by fragmenting peptide chains, cross‐linking proteins and adding carbonyl groups, which renders proteins more susceptible to proteolytic degradation. Such modifications can inhibit enzymatic activity, increase the propensity for protein aggregation and proteolysis, alter cellular uptake mechanisms and modify immunogenic properties. NOX enzymes are involved in a range of critical biological processes, including cellular signalling, metabolism, stress responses, host defence and the maintenance of tissue homeostasis [22, 23].

There is increasing evidence linking NOX enzymes to neurobehavioral disorders, particularly neurodegenerative conditions such as Alzheimer disease and related dementia [23, 24, 25], also Parkinson's disease dementia [26, 27]. Microglia and astrocytes are key mediators of age‐related neuroinflammation. Enhanced microglial activation is associated with the generation of excessive ROS, which can exacerbate oxidative damage within the central nervous system (CNS). In neurodegenerative diseases, however, these microglia can become ‘disease‐associated’, meaning they take on a pro‐inflammatory and harmful role that can further contribute to neuronal damage [28].Activation of NOX enzymes in microglia and astrocytes contributes to the production of ROS during neuroinflammatory responses. In the context of injury, activated microglia and astrocytes generate elevated levels of ROS through NOX enzymes, which adversely affect the expression of molecules that regulate the integrity of the blood–brain barrier (BBB). This disruption of BBB function is implicated in the pathogenesis of neurodegeneration and cognitive decline, underscoring the role of NOX‐mediated oxidative stress in neuroinflammatory and neurodegenerative diseases [23, 29]. The activation of NOX by p‐cresol has been recorded in mouse 3T3‐L1 adipocytes [20]. More investigations will be necessary to verify whether this interesting result can be replicated and extended to other cell lines and species, including humans.

BDNF is a neurotrophin predominantly expressed in the hippocampus and cerebral cortex, where it plays a critical role in neuronal development, synaptic plasticity, learning, memory, mood regulation, neurogenesis and neuroprotection [20, 30]. BDNF exerts its effects through its mature form, which promotes neuronal survival and plasticity. However, the precursor form, pro‐BDNF, can trigger apoptosis, reduce dendritic spine density and contribute to depressive‐like states, particularly within the hippocampus. Dysregulation of BDNF signalling, potentially induced by compounds such as p‐cresol, may impair BDNF production and disrupt its associated signalling pathways. Such dysregulation has been implicated in brain atrophy, cognitive decline and an elevated risk of psychiatric disorders [31, 32].

The study investigated the impact of p‐cresol on BTBR mice, a model for ASD. Mice were maintained under controlled conditions with ad libitum access to food and water and a 12:12‐h light–dark cycle, with behavioural testing conducted between postnatal days 60–70. Following a single acute injection of p‐cresol, behavioural assessments commenced 15 min later, alongside biochemical analyses of brain tissue to evaluate neurotransmitter levels and metabolites. The findings demonstrated that p‐cresol administration induced behavioural abnormalities that mimic core ASD symptoms, including heightened anxiety‐like behaviours, hyperactivity and stereotypic actions. The study underscores the importance of gene–environment interactions, highlighting how genetic susceptibility interacts with environmental exposure to neurotoxic agents such as p‐cresol. Moreover, it was found that p‐cresol disrupts dopaminergic activity in the brain, particularly in the amygdala and striatum, which are critical for reward processing and social behaviours [33], prefrontal cortex of susceptible mice [33, 34], decreased activity of central dopamine neurons involved in the social reward circuit [35], and also abnormal dopamine metabolism in autism [36]. In addition to the previously shown disruption of dopaminergic axis [35, 37], also serotonergic neurotransmission may be negatively influenced by p‐cresol and PCS [13]. The majority of serotonin is stored intracellularly within synaptic vesicles, from which it is released into the synaptic cleft upon neuronal depolarization. Once released, serotonin binds to specific receptors located on both presynaptic and postsynaptic membranes, influencing diverse physiological functions and behaviour. Dysregulation of serotonin production and release, potentially induced by p‐cresol, can have profound negative effects also on both physiological processes and behavioural outcomes, contributing to mood disorders, cognitive impairment and other neuropsychiatric conditions [31].

EVs are membrane‐bound particles derived from various cellular compartments and processes, which are released into the extracellular space. EVs play a critical role in intercellular communication, facilitating the transfer of bioactive molecules between cells. These vesicles possess the capacity to deliver antioxidants to adjacent cells, thereby acting as scavengers of ROS, while concurrently carrying enzymes involved in ROS production. The functional characteristics of EVs, including their ability to modulate oxidative stress, are influenced by the physiological and pathological conditions, as well as the redox status, of the cells from which they originate. p‐Cresol has been shown to enhance the formation of EVs, which, in turn, can promote ROS generation, contributing to the induction of oxidative stress [38].

Transcription factors (TFs) are crucial regulators of gene expression, controlling essential cellular processes such as proliferation, survival, self‐renewal and invasion. CREB, a key cellular transcription factor, mediates cellular responses to various physiological and pathological signals. In the context of oxidative stress, ROS induces the activation of CREB, which plays a pivotal role in promoting cell survival. CREB regulates the expression of several genes involved in ROS detoxification, including the upregulation of the APE1 gene and the induction of anti‐apoptotic genes. Studies have demonstrated that exposure to p‐cresol leads to an increase in CREB levels, highlighting the cellular response to enhance cell survival in the face of oxidative stress. The overexpression of CREB activates DNA damage response genes, facilitating DNA repair mechanisms and preventing apoptosis. This adaptive response underscores the critical role of CREB in maintaining cellular integrity under conditions of oxidative stress [39].

**FIGURE 2:**
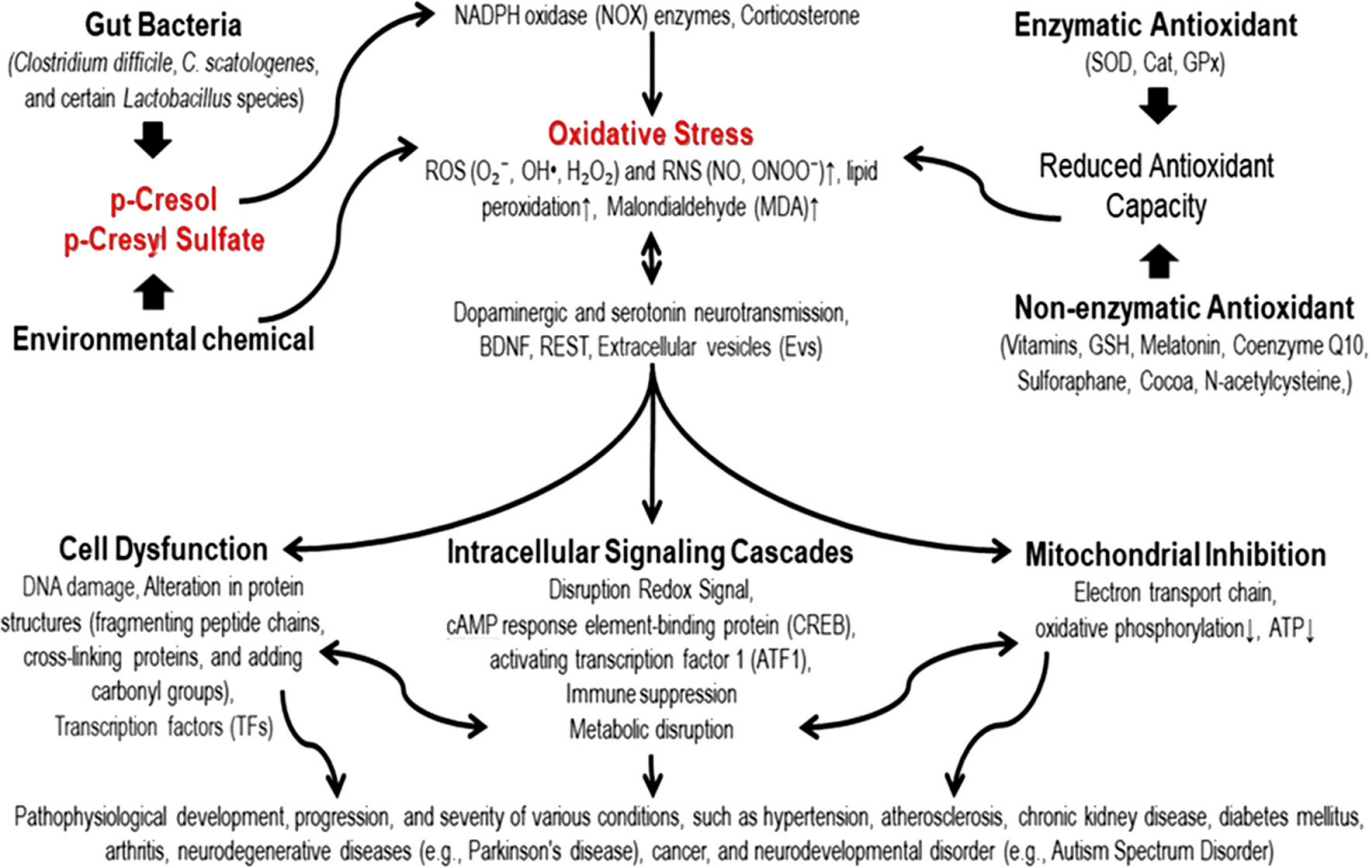
Mechanisms underlying p‐cresol‐ and p‐cresyl sulphate‐induced oxidative stress, and its negative consequences.

Based on the findings from the reviewed studies, several clinical implications of p‐cresol and its derivatives can be identified. p‐Cresol and PCS boost oxidative stress, contribute to cellular dysfunction, disrupted intracellular signalling cascades and mitochondrial inhibition, thereby exacerbating the pathophysiological development, progression and severity of various conditions, including hypertension, atherosclerosis, chronic kidney disease, diabetes mellitus, arthritis, neurodegenerative diseases (e.g., Parkinson's disease), cancer and neurodevelopmental disorders (e.g., autism spectrum disorder), as illustrated in Figure 2. Future therapeutic strategies for diseases associated with oxidative stress may involve targeting p‐cresol and its metabolites with potential therapeutic agents able either to reduce gut absorption or minimize production, for example by limiting the proliferation of cresol‐producing clostridia with appropriate prebiotics. Additionally, the measurement of p‐cresol and PCS levels performed in parallel with peripheral measures of oxidative stress could serve to monitor longitudinally p‐cresol contributions to diseases associated with excessive oxidative stress. To mitigate the oxidative stress induced by p‐cresol and PCS, interventions such as antioxidants and enzyme inhibitors may offer potential therapeutic avenues for reducing the detrimental effects of these compounds.

Previous studies primarily focused on p‐cresol and PCS roles as uremic toxins in CKD and their ability to precipitate ROS, leading to systemic oxidative damage, inflammation [40] and inhibit endothelial repair which participates in accelerated atherosclerosis, is a hallmark of CKD patients and make them at higher risk of cardiovascular diseases than the general population [41, 42].

New studies have demonstrated that p‐cresol directly disrupts dopaminergic activity in brain regions critical for social and reward processing, such as the amygdala and striatum, linking its oxidative effects to specific behavioural outcomes, including hyperactivity, anxiety and impaired social interaction in ASD models [19, 33, 40]. Moreover, advancements in biochemical techniques have uncovered that PCS exacerbates mitochondrial dysfunction, leading to impaired ATP production and amplified ROS generation [20, 43].

The recent focus on gene–environment interactions further underscores how environmental exposure to these compounds interacts with genetic predispositions to influence the severity and progressivity of disease such as ASD pathophysiology and neurodegenerative diseases [26, 44].

The strengths of the studies included in this review lie in the robustness of their experimental designs, notably the use of multiple model systems to assess the effects of p‐cresol on oxidative stress, as well as the consistency of the findings across studies, which collectively support the involvement of p‐cresol in promoting oxidative stress, at least at the high blood concentrations typically encountered in uremic patients (100–250 mM). However, several limitations are also inherent to these six studies. These include variability in experimental methodologies, such as differences in p‐cresol concentrations, experimental conditions and outcome measurements, which may affect the comparability and generalizability of results. Additionally, the lack of long‐term in vivo studies or clinical trials limits the ability to assess the chronic effects and clinical relevance of p‐cresol exposure. Furthermore, the potential for publication bias must be considered, as studies with positive results are more likely to be published, which could skew the overall findings.

Given these limitations, it is imperative that further research be conducted to elucidate the precise molecular mechanisms underlying the effects of p‐cresol and p‐cresyl sulphate (PCS) on oxidative stress. Additionally, studies should investigate the long‐term consequences of elevated p‐cresol concentrations in the body, particularly with regard to CKD, neurodegenerative and neurodevelopmental disorders, as well as its potential role in disease progression. The establishment of standardized experimental protocols is also recommended to enhance the comparability and reproducibility of findings across studies, thereby facilitating more robust conclusions in future research.

## 5 Conclusion

In summary, this review of the most recent literature shows that p‐cresol and PCS are involved in boosting oxidative stress pathways through several mechanisms, activation of NADPH oxidase, dysregulation of neurotrophic factors and neurotransmitters, and the induction of extracellular vesicle (EV) formation. These results spur interest into therapeutic strategies aimed at reducing p‐cresol production (prebiotics) and absorption (selective cresol‐binding adsorbents like divinylbenzenic resin) [21, 45] in addition to more traditional antioxidants, which some in vitro studies presented here do find effective in limiting the damage produced by cresol‐ and PCS‐induced oxidative stress, at least in vitro. Although more research is necessary to shed light on the complex set of molecular pathways underlying cresol‐induced oxidative stress, these recent studies provide converging lines of evidence that indeed increased oxidative stress is one of the main mechanisms involved in cellular and tissue damage produced by p‐cresol and its derivatives. Its prevention and correction thus merit to be further explored also for their potential clinical benefits.

## Conflicts of Interest

The authors declare no conflicts of interest.

## Supporting information


**Supplementary Table 1A.** Newcastle–Ottawa Quality Assessment Scale for case–control studies.
**Supplementary Table 1B.** Joanna Briggs Institute Critical Appraisal Tool for in vitro quasi experimental studies.

## Data Availability

Data sharing is not applicable to this article as no new data were created or analysed in this study.
