# Supplementary material for: p‐Cresol and p‐Cresyl Sulphate Boost Oxidative Stress: A Systematic Review of Recent Evidence
Source: Basic Clin Pharmacol Toxicol. 2025 Jun 18;137(1):e70065. doi: 10.1111/bcpt.70065 (PMC12177445; doi:10.1111/bcpt.70065)
Supplement: Supplementary file 1 — Supplementary Table 1A. Newcastle–Ottawa Quality Assessment Scale for case–control studies. Supplementary Table 1B. Joanna Briggs Institute Critical Appraisal Tool for in vitro quasi experimental studies. [file BCPT-137-0-s001.docx]

**Supplementary Material**

**p-Cresol and p-Cresyl Sulfate Boost Oxidative Stress:**

**A Systematic Review of Recent Evidence**

Rinvil Renaldi,^1,2,3^ Tjhin Wiguna,^4,5^ Antonio M. Persico,^6,7^ Andi Jayalangkara Tanra^3^

^1^Doctoral Program, Faculty of Medicine, Hasanuddin University, Makassar, Indonesia.

^2^Child and Adolescent Division, Department of Psychiatry, Faculty of Medicine, Hasanuddin University, Makassar, Indonesia.

^3^Department of Psychiatry, Faculty of Medicine, Hasanuddin University, Makassar, Indonesia.

^4^Child and Adolescent Division, Department of Psychiatry, Faculty of Medicine, University of Indonesia, Jakarta, Indonesia.

^5^Department of Psychiatry dr. Cipto Mangunkusumo General Hospital, Jakarta, Indonesia

^6^Department of Biomedical, Metabolic and Neural Sciences, University of Modena and Reggio Emilia, Modena, Italy.

^7^Child & Adolescent Neuropsychiatry Program, Modena University Hospital, Modena, Italy.

**Running title:** p-Cresol and oxidative stress

**Keywords:** autism spectrum disorder, chronic kidney disease, gastrointestinal microbiome, Parkinson disease, uremic toxins

**Correspondence:** Antonio M. Persico, Department of Biomedical, Metabolic and Neural Sciences, University of Modena and Reggio Emilia, Modena, Italy.

email: antonio.persico@unimore.it

**Funding Information:** R.R has received funding through the Center for Higher Education Funding and Assessment, Ministry of Higher Education, Science, and Technology of the Republic of Indonesia. The funder had no role in study design, data collection, analysis, interpretation, or writing of the manuscript.

**Supplementary Table 1A.** Newcastle-Ottawa Quality Assessment Scale for case-control studies.


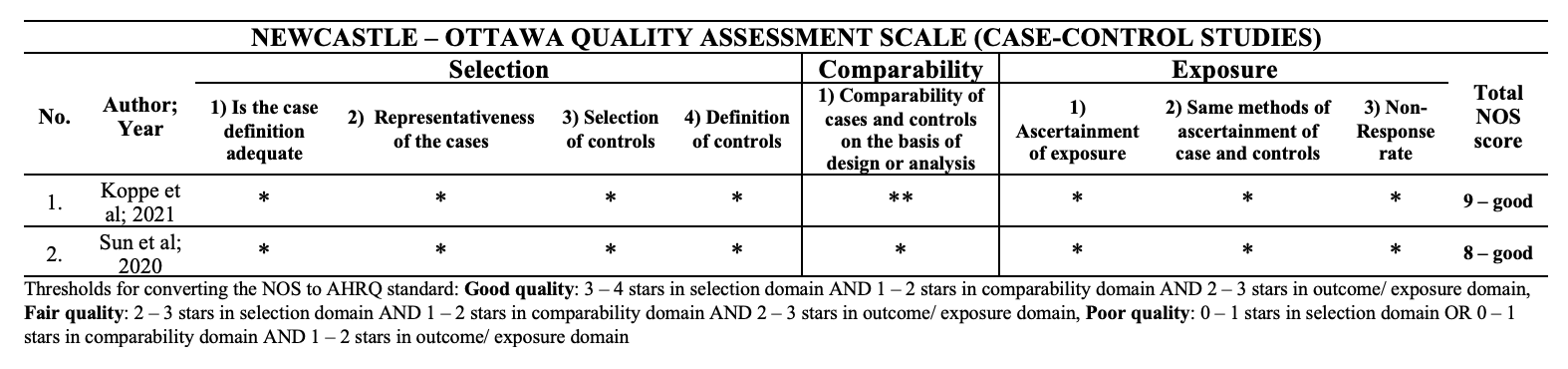


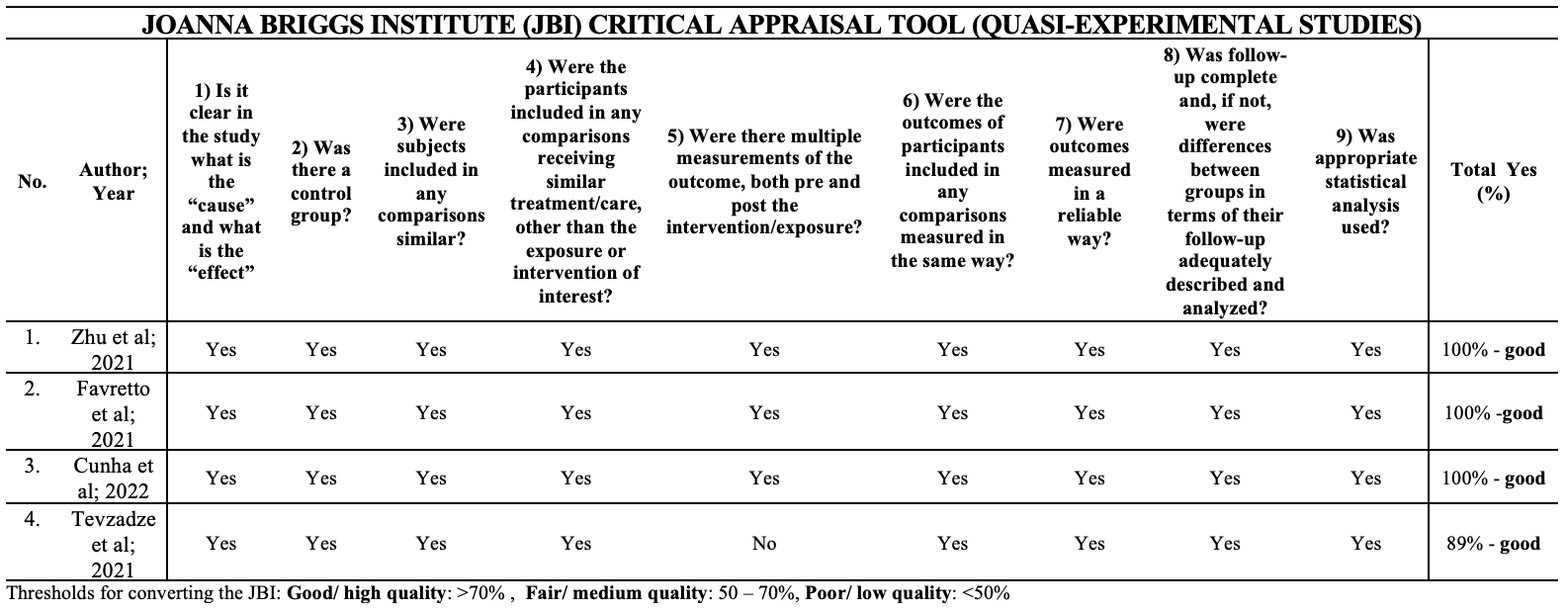
**Supplementary Table 1B.** Joanna Briggs Institute Critical Appraisal Tool for in vitro quasi experimental studies.
